# The Intraseptal Course of the Superficial Peroneal Nerve: An Anatomic Study

**DOI:** 10.1177/10711007211002508

**Published:** 2021-06-19

**Authors:** Silvia Valisena, Axel Gamulin, Didier Hannouche

**Affiliations:** 1 Service de Chirurgie Orthopédique et Traumatologie de l’appareil locomoteur Hôpitaux Universitaires de Genève, Geneva, Switzerland

**Keywords:** superficial peroneal nerve, anatomic variant, intraseptal variant, fascial tunnel, nerve injury

## Abstract

**Background:**

Anatomic and clinical studies show many variants of the superficial peroneal nerve (SPN) course and branching within the compartments and at the suprafascial layer. The anatomy of the transition zone from the compartment to the subcutaneous layer has been occasionally described in the literature, mainly in studies reporting the intraseptal SPN variant in 6.6% to 13.6% of patients affected by the SPN entrapment syndrome. Despite the little evidence available, the knowledge of the transition zone is relevant to avoid iatrogenic lesions to the SPN during fasciotomy, open approaches to the leg and ankle, and SPN decompression. Our anatomic study aimed to describe the SPN transition site and to evaluate the occurrence of a peroneal tunnel and of an intraseptal SPN variant.

**Methods:**

According to the institutional ethics committee requirements, 15 fresh-frozen lower limbs were dissected to study the SPN course and its branching, focusing on the transition site to the suprafascial layer.

**Results:**

The SPN was located in the anterior compartment in 2 cases and in the lateral in 13. An intraseptal tunnel was present in 10 legs (66%), at a mean distance of 10.67 cm from the lateral malleolus. Its mean length was 2.63 cm. The tunnel allowed the passage of the main SPN in 8 cases and of its branches in two. In the remaining 5 legs (33%), the SPN pierced a crural fascia window.

**Conclusion:**

In our sample a higher rate than expected of intraseptal SPN variants was found.

**Clinical Relevance:**

The knowledge of the anatomy of the SPN course and intraseptal variant is relevant to avoid iatrogenic lesions during operative dissection. Further studies are needed to evaluate the effective prevalence of an intraseptal tunnel, independently from the SPN entrapment syndrome, and how to avoid associated iatrogenic complications.

## Introduction

The superficial peroneal nerve (SPN) branches out from the common peroneal nerve within the peroneus longus muscle in the lateral compartment of the leg, distal to the proximal peroneal metaphysis.^11^ According to most of the anatomy textbooks,^11^ the SPN courses in the lateral compartment, across the peroneus longus and peroneus brevis muscles, giving them motor branches. It pierces the crural fascia at the distal third of the leg and distally bifurcates in the medial and intermediate dorsal cutaneous nerves (respectively, MDCN and IDCN) to innervate the dorsal aspect of the foot.^8^ The literature shows much variability of the SPN course and branching at the intracompartmental and suprafascial sites. Anatomic and clinical studies have shown that the SPN lies in the lateral compartment in 57% to 73% of cases, in the anterior in 8.1% to 23%, and branches in both of them in 5% to 26%.^1,3,6,7,10^ A meta-analysis on the SPN variability showed a suprafascial course with a distal bifurcation in MDCN and IDCN in 82.7% of cases (type 1), an intracompartmental branching in MDCN and IDCN superficializing as separate entities in 15.6% (type 2), and the absence of IDCN in 1.8% (type 3).^16^ According to the SPN branching type, the distance between the SPN suprafascial emergence site and the apex of the lateral malleolus or the intermalleolar line varies between the middle and distal third of the fibula.^1,4,7,12,17^

Nevertheless, the description of the transition site from the intracompartmental to the suprafascial level has been rarely reported in the literature and can be mainly found in clinical studies describing the SPN entrapment syndrome, a condition affecting 3.5% of patients with chronic leg pain.^6,10,13,14,18^ In these cases, at the transition site, the SPN coursed into a fibrous tunnel described as “intraseptal” or “peroneal” tunnel, whose length ranged from 3 to 11 cm.^13,14,18^ Here, the SPN was compressed either by the fibrotic tissue of the tunnel or by a muscle herniation through a fascial defect, requiring operative decompression to relieve the entrapment symptoms.^13,14,18^

The anatomy of the SPN transition site has clinical and operative relevance not only for the SPN entrapment syndrome, but also for several procedures requiring SPN identification and sparing, such as anterior and lateral compartments’ fasciotomies, and anterior, anterolateral, and lateral approaches to the ankle. The present anatomic study aimed to describe the characteristics of the SPN transition site from the compartment to the suprafascial site and to describe the features of any peroneal tunnel and intraseptal SPN variant.

## Materials and Methods

The study, which received ethical approval by the local Ethics Committee (study number 2020-02643, Commission cantonal d’éthique de la recherche de Genève), was conducted on 15 fresh-frozen lower limbs, 9 right and 6 left, from 9 cadavers of adult donors. The Institute of Anatomy provided only the age range of donors (76-94 years) to avoid the generation of identifiable information. The subjects were 6 women and 3 men, whose mean height was 163.5 cm.

Only donors without evidence of ankle surgery were considered eligible. The limbs were placed in a supine position, slightly in internal rotation. The skin incision started proximally and posterolaterally above the knee joint, and extended distally along the fibula, following the projection of the anterior intermuscular septum (AIS). The crural fascia was longitudinally incised both on the anterior and lateral compartments, 1 cm lateral to the tibial crest and posterior to the peroneus longus tendon, respectively. The SPN was located and dissected distally starting from its origin from the common peroneal nerve. The location, course, and branches of the intracompartmental SPN were recorded. Distally, the MDCN and the IDCN were located in the subcutaneous tissue, superficially relative to the crural fascia, anteriorly relative to the fibula, at the level of the tibiotalar joint. Once located, the branches were dissected proximally to locate their origin on the SPN. The relationships between the SPN, the MDCN, and the IDCN and the AIS at the transition site were recorded. In the case of an intraseptal tunnel, its length and the distance of its deep and superficial opening from the apex of the lateral malleolus were measured. The distal insertion of the AIS was measured from the apex of the lateral malleolus and the superficial opening of an intraseptal tunnel. The superficial branching of the SPN was described according to Takao et al,^15^ and the SPN bifurcation in MDCN and IDCN was measured from the lateral malleolus apex. The fibular length was recorded.

The study involved a descriptive analysis of the SPN and all its superficial nerve rami and their relationships with the AIS and the lateral malleolus. For all the measurements, the absolute and percentage value was reported, and the mean and standard deviation were calculated.

## Results

### Superficial Peroneal Nerve Distribution

The SPN was found in the lateral compartment in 13 cases, and in the anterior compartment in 2 cases. The division of the SPN in MDCN and IDCN was at the suprafascial layer in 12 cases and intracompartmental in 3. In the latter, the SPN divided proximally in the leg, as shown in Table 1.

**Table 1.** Demographics, Nerve Course, and Branching Description With Measurements at the Ankle.

Leg Number (Case Number)	Side	Gender	Height, cm	Compartment	MDCN and IDCN Bifurcation Site,^a^ Proximal to LM, cm	Suprafascial Branches: Takao Classification^15^
1 (1)	R	F	153	MN: lateral MDCN: s-f IDCN: s-f	11.5	3
2 (2)	R	F	163	MN: lateral MDCN: s-f IDCN: s-f	8	2
3 (2)	L	F	163	MN: lateral MDCN: s-f IDCN: s-f	3.5	2
4 (3)	L	F	172	MN: lateral MDCN: s-f IDCN: s-f	Distal to LM	1
5 (3)^b^	R	F	172	MN: lateral MDCN: lateral IDCN: lateral	32.5	ICB
6 (4)	L	M	173	MN: lateral MDCN: s-f IDCN: s-f	7.3	3
7 (4)	R	M	173	MN: lateral MDCN: anterior IDCN: lateral	31	ICB
8 (5)	R	F	152	MN: anterior MDCN: s-f IDCN: s-f	3.5	2
9 (6)	R	F	150	MN: anterior MDCN: s-f IDCN: s-f	Distal to LM	1
10 (7)	L	F	156	MN: lateral MDCN: s-f IDCN: s-f	3.5	2
11 (7)	R	F	156	MN: lateral MDCN: lateral IDCN: lateral	15.5	ICB
12 (8)	L	M	178	MN: lateral MDCN: s-f IDCN: s-f	8	3
13 (8)	R	M	178	MN: lateral MDCN: s-f IDCN: s-f	8.3	3
14 (9)	L	M	175	MN: lateral MDCN: s-f IDCN: s-f	7.5	2
15 (9)	R	M	175	MN: lateral MDCN: s-f IDCN: s-f	11.2	2
Overall		Total, n (%)	Mean (SD)	Total, n (%)	Mean (SD)	Total (%)
	R: 9 legs L: 6 legs	F: 9 legs (60), 6 cases (66). M: 6 legs (40%), 3 cases (33%).	163.5 cm (11.10)	AC: 2 MN (13), 1 MDCN (6). LC: 13 MN (86), 2 MDCM (13), 3 IDCN (20). s-f: 12 MDCN (80), 12 IDCN (80).	11.63 cm (9.56) Mean s-f: 7.23 cm (2.94) Mean ICB: 26.3 cm (9.41)	Type 1: 2 legs (13%) Type 2: 6 legs (40%) Type 3: 4 legs (26%) ICB: 3 legs (20%)

Abbreviations: AC, anterior compartment; F, female; ICB, intracompartmental bifurcation; IDCN, intermediate dorsal cutaneous nerve; L, left; LC, lateral compartment; LM, tip of the lateral malleolus; M, male; MCDN, medial dorsal cutaneous nerve; MN, main nerve; R, right; s-f, suprafascial.

a MDCN and IDCN bifurcation site: distance of the SPN bifurcation in MDCN and IDCN from the apex of the lateral malleolus.

b The accessory deep peroneal nerve was described in the lateral compartment of the specimen 5.

At the transition site, the SPN penetrated the septum and coursed into an intraseptal tunnel in 10 cases (66%; Table 2). The branch passing through the tunnel was the SPN in 8 cases (53%), arising from the lateral compartment in 6 cases, and the anterior compartment in 2 cases. In one case, the IDCN left the lateral compartment passing in the intraseptal tunnel (leg 11). In another, the MDCN and the IDCN branched from the SPN in the lateral compartment, and both nerves ran within 2 different tunnels in the septum (leg 5).

**Table 2.** Features of the Transition Site.

Leg Number (Case Number)	Intraseptal	Deep Opening of the Tunnel: Distance From LM,^a^ cm (Ratio)	Superficial Opening of the Tunnel: Distance From LM,^b^ cm (Ratio)	Anterior Septum Ending: Distance From the Superficial Opening, cm	Anterior Septum Ending: Distance From LM, cm	Tunnel Length, cm	Fibular Length, cm
1 (1)	MN	13.7 (2.51)	12.5 (2.76)	3.5	9	1.2	34.5
2 (2)	No	9.5 (3.78)	9.5 (3.78)	0.5	9	0	36
3 (2)	MN	13 (2.73)	9.3 (3.81)	3.8	5.5	3.7	35.5
4 (3)	MN	12 (3.29)	10.7 (3.69)	2	8.7	1.3	39.5
5 (3)^c^	MDCN and IDCN	MDCN: 19.5 (2.03) IDCN 10.3 (3.84)	MDCN: 17 (2.32) IDCN: 6.5 (6.09)	MDCN: 8.8 IDCN: 2.1	8.2	MDCN: 2.5 IDCN: 3.8	39.6
6 (4)	No	11.3 (3.36)	11.3 (3.36)	2.3	9	0	38
7 (4)	No	MDCN: AC 14.4 (2.65) IDCN: LC 8.8 (4.35)	MDCN: AC 14.4 (2.65) IDCN: LC 8.8 (4.35)	MDCN 6.1 IDCN: 0.5	8.3	MDCN: AC 0 IDCN: LC 0	38.3
8 (5)	MN	14.2 (2.5)	11.2 (3.16)	2.7	8.5	3	35.5
9 (6)	MN	12 (2.75)	11 (3.3)	1	10	1	33
10 (7)	MN	10 (3.6)	6 (6)	2	8	4	36
11 (7)	IDCN	11 (3.27)	7 (5.14)	1.5	8.5	4	36
12 (8)	No	11 (3.59)	11 (3.59)	2.3	8.7	0	39.5
13 (8)	MN	11(3.54)	8.5 (4.58)	0.3	8.8	2.5	39
14 (9)	MN	16 (2.5)	14 (2.85)	7	7	2	40
15 (9)	No	12.7 (3.14)	12.7 (3.14)	4.2	8.5	0	40
Overall	Total, n (%)	Mean (SD)	Mean (SD)	Mean (SD)	Mean (SD)	Mean (SD)	Mean (SD)
	Intraseptal: 10 (66) MN: 8 (53) MDCN: 1 (6) IDCN: 2 (13) Extraseptal: 5 (33)	Overall: 12.37 cm (2.64) Intraseptal: 12.97 cm (2.81) Extraseptal: 11.28 cm (2.05) Overall ratio: 3.14 (0.61) Intraseptal ratio: 2.96 (0.57) Extraseptal ratio: 3.47 (0.57)	Overall: 10.67 cm (2.93) Intraseptal: 10.33 cm (3.36) Extraseptal: 11.28 cm (2.05) Overall ratio: 3.79 (1.10) Intraseptal ratio: 3.97 (1.30) Extraseptal ratio: 3.47 (0.57)	Overall: 2.97 cm (2.39) Intraseptal: 3.15 cm (2.58) Extraseptal: 2.65 cm (2.18)	8.38 cm (1.02)	2.63 cm (1.15)	37.36 cm (2.26)

Abbreviations: AC, anterior compartment; IDCN: intermediate dorsal cutaneous nerve; LC, lateral compartment; LM, tip of the lateral malleolus; MCDN, medial dorsal cutaneous nerve; MN, main nerve.

a Distance from LM described as ratio of the fibular length to the deep opening of the tunnel.

b Distance from LM described as ratio of the fibular length to the superficial opening of the tunnel.

c One intraseptal tunnel for the MDCN and one for the IDCN.

In the remaining 5 cases, the main nerve or its branches crossed a crural fascia window to reach the subcutaneous tissue (33%, here called extraseptal cases). In one extraseptal case, the SPN divided in its branches in the lateral compartment, then the MDCN coursed in the anterior and the IDCN in the lateral, before penetrating the subcutaneous tissue through a separate crural fascia window (leg number 7). One case of accessory deep peroneal nerve was described (Table 1).

The distribution of the suprafascial branches was described according to Takao classification,^15^ except for 3 legs with intracompartmental branching (Table 1). In most of the cases (6 legs, 40%) the MDCN and IDCN branched from the SPN at, or proximal to the ankle and subsequently divided in their distal branches at the Chopart or Lisfranc level, as described in Takao type 2.

As shown in Tables 1 and 2, the anatomy was not symmetric in cases who underwent the dissection of both legs.

### Intraseptal Tunnel

An intraseptal tunnel was present in 10 legs, allowing the passage of the main SPN in 8 cases and of its branches in the remaining 2 cases (Table 2). The mean intraseptal tunnel length was 2.63 cm (range 1-4 cm).

The nerve superficialized 10.67 cm proximal to the lateral malleolus (overall mean). In the intraseptal cases, the nerve arose from the superficial tunnel opening at a mean 10.33 cm from the lateral malleolus apex, whereas it arose slightly more proximal for the extraseptal cases (mean 11.28 cm).

Overall, the AIS ended at a mean 8.38 cm (range 5.5-10 cm) from the lateral malleolus and 2.97 cm (range 0.3-8.8 cm) distal to the suprafascial emergence of the nerve.

The mean fibular length was 37.36 cm. The distance between the superficialization site and the apex of the lateral malleolus was adjusted for the fibular length, to obtain a ratio measuring this distance as a proportion of the fibular length. In average, the nerve superficialized between the distal two-fourths of the fibula (mean of the ratios 3.79).

### Transition Zone in Intraseptal Cases

**Figure 1.**
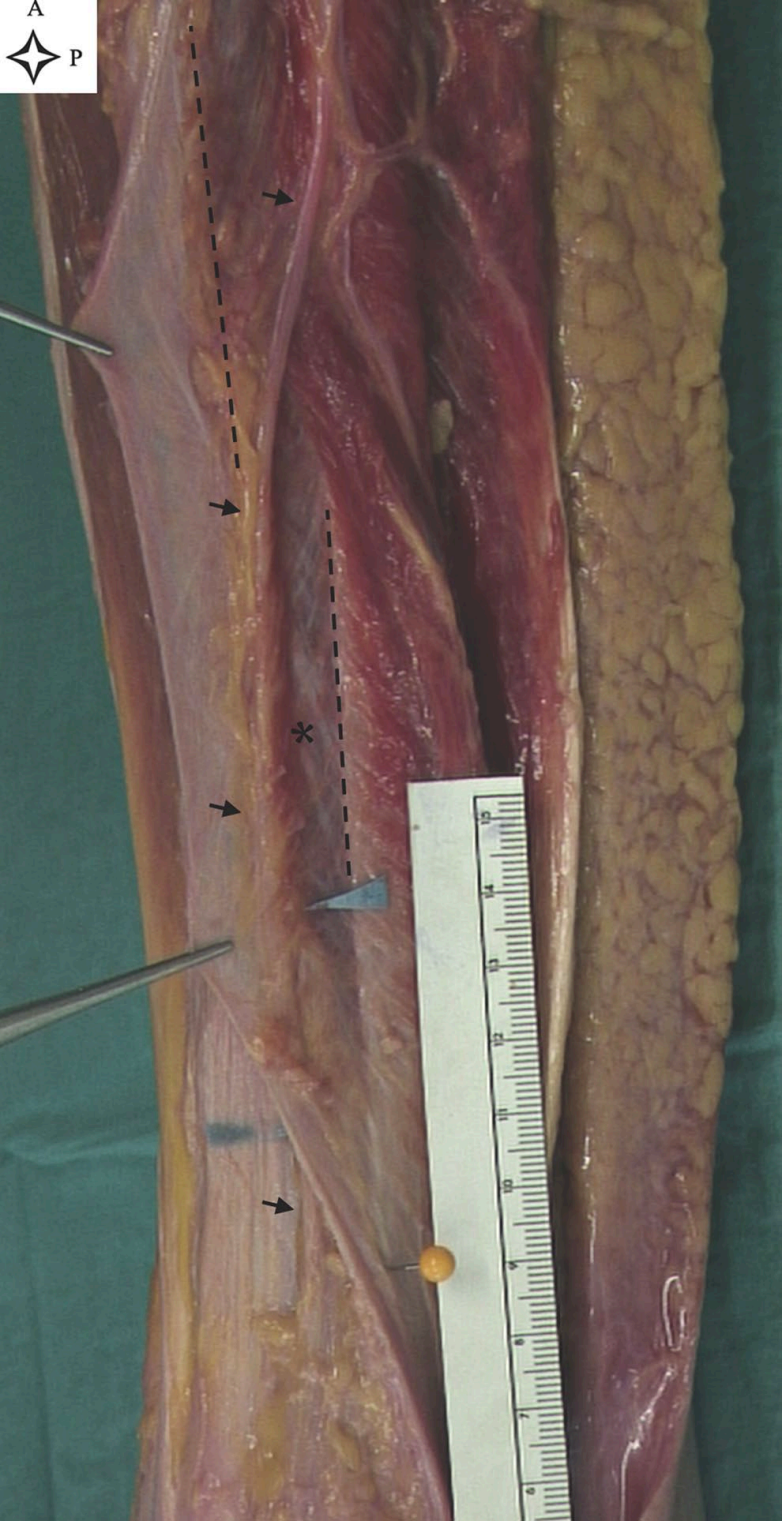
Superficial peroneal nerve course in the lateral compartment (leg 4, left leg). The forceps are holding the crural fascia. Black arrows show the superficial peroneal nerve, within the lateral compartment and suprafascial layer. Blue arrows show the nerve proximal and distal to the deep and superficial opening of the intraseptal tunnel. The dotted lines show the upper and lower border of the anterior intermuscular septum (AIS). The yellow needle shows the distal insertion of the AIS on the fibula. The asterisk indicates the muscle fibers and connective tissue tethering the nerve to the AIS; note the thick fibers of the septum. A, anterior; P, proximal.

**Figure 2.**
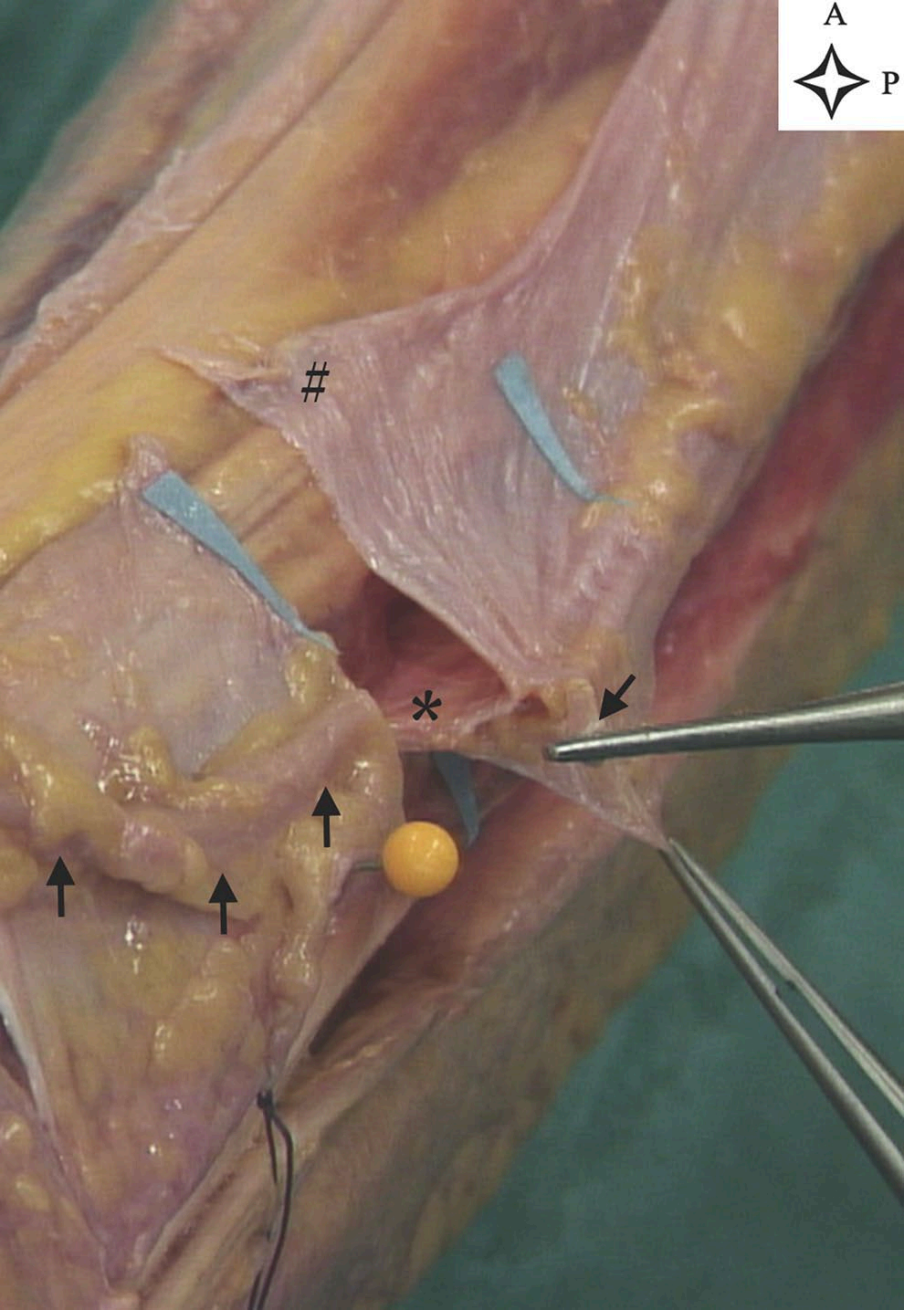
Section of the tunnel showing the Y structure (leg 4, left leg). The tunnel was cut at its distal opening. Blue paper arrows show the distal and proximal tunnel opening. Black arrows show the superficial peroneal nerve. The upper forceps are holding the nerve and the lower forceps the crural fascia. The asterisk shows the anterior intermuscular septum. The hash sign indicates the crural fascia. A, anterior; P, proximal.

**Figure 3.**
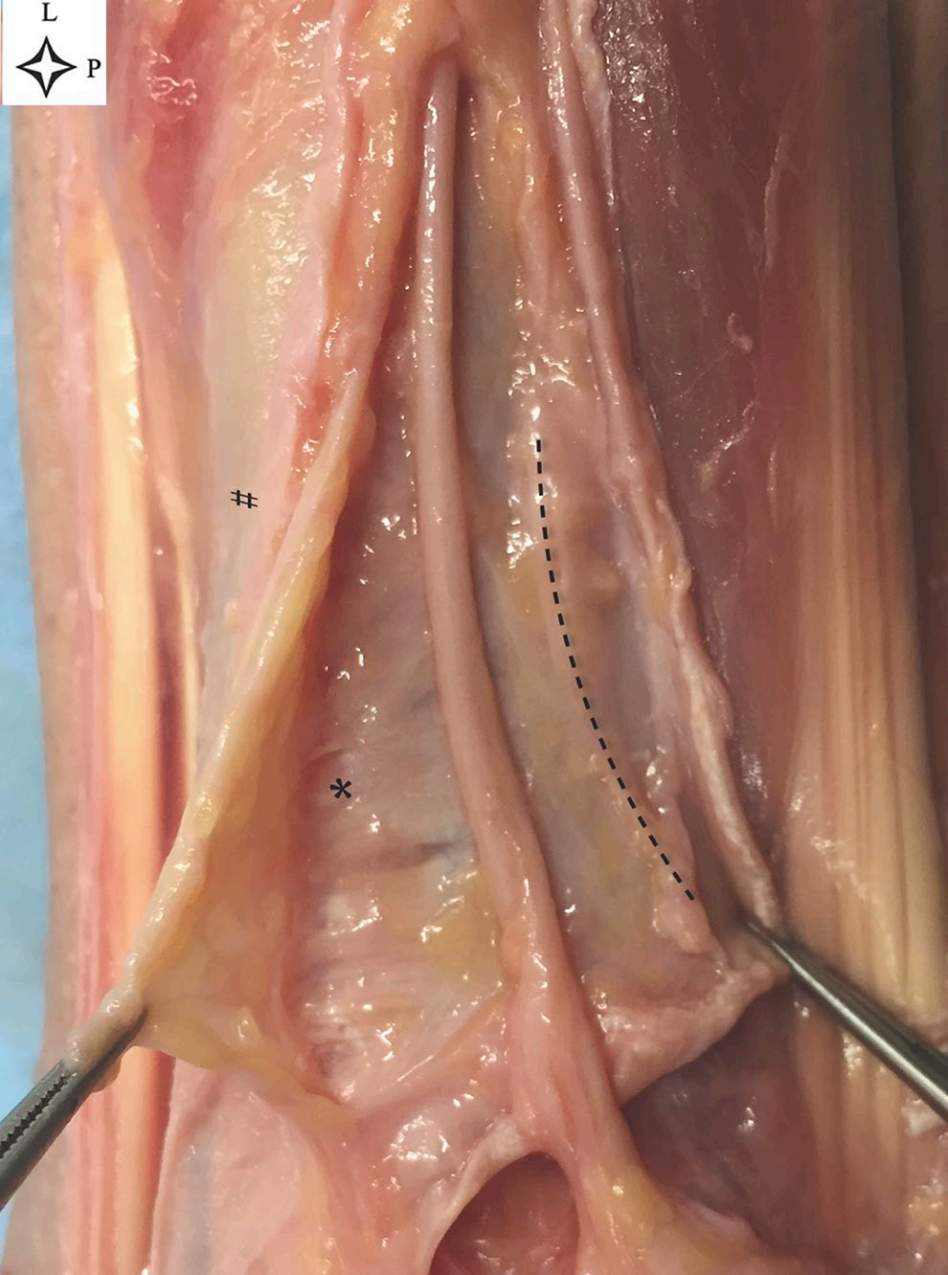
Roof opening and nerve exposure (leg 13, right leg). The upper forceps are holding part of the tunnel’s roof, and the lower forceps can be seen holding the crural fascia. The dotted line marks the the folded-over inferior half of the roof. The asterisk indicates the thick fibers at the base of the tunnel. The hash sign shows the crural fascia. Note the difference fibers pattern between crural fascia, roof of the tunnel, base of the tunnel. L, lateral; P, proximal.

At the lateral compartment, the SPN passed through the peroneus longus and peroneus brevis muscle or within extensor digitorum longus or peroneus tertius muscle in the case of an anterior compartment course. Then, it reached the AIS and descended laterally to this structure, until the interface between the superior margin of the AIS and the deeper aspect of the crural fascia (Figure 1). Here, the nerve was tethered to the AIS by some muscular fibers and protected by a layer of fat tissue. The SPN entered a Y-shaped fibrous structure (Figure 2), where the lower head was the AIS, and the higher prongs the walls of the intraseptal tunnel.^14,18^ The nerve was enveloped in the tunnel, surrounded by a layer of fat and connective tissue. Macroscopically, the thick fibrous fibers characterizing the AIS were less densely represented at the tunnel’s walls and absent at its roof, which presumably originated from the crural fascia (Figures 1 and 3).

From an intracompartmental view, the passage between the AIS and the deep fascia was net, not allowing the visual distinction of an intraseptal tunnel (Video, leg 4). Once the tunnel roof incised, the SPN appeared surrounded by protective fat tissue. The transposition of the nerve showed the thick fibrous base of the tunnel, consisting of the superior border of the AIS, and thin lateral walls, originating from the margins of the AIS and in continuity with the crural fascia constituting the tunnel roof and walls (Figure 3). The roof and the walls were thin and elastic, compared to the thick and fibrous AIS.

## Discussion

Our anatomic study aimed to evaluate how the SPN reaches the suprafascial layer, describing the characteristics of its passage in the transition zone and any intraseptal SPN variant. Here we showed that the nerve crossed a crural fascia window in 5 cases and passed through an intraseptal tunnel in 10, with a prevalence of intraseptal SPN variant of 66% (10/15 legs).

The peroneal tunnel and intraseptal SPN variant are described in studies on the SPN entrapment^13,14,18^ and in one clinical and anatomic study^6^ evaluating the SPN branching pattern. The prevalence of the intraseptal SPN variant reported in the case of the entrapment syndrome ranges from 13.6% to 58% of the operated legs,^13,14,18^ whereas it comprised 6.3% in a study including also patients operated for traumas as well as anatomic specimens.^6^ The exact prevalence of intraseptal variants is difficult to determine in the literature, as it might be overestimated in clinical studies focusing on SPN entrapment and underestimated in anatomic studies, which mainly focused on the branching of the SPN.

In his clinical studies, Styf et al^13,14^ hypothesized that short (<3-cm) tunnels should be considered as a normal finding unless they are associated with a fascial defect or a muscular hernia, which could be responsible for an entrapment syndrome. In contrast, he observed that long (3-11 cm) fibrotic tunnels were more frequently associated with an entrapment syndrome and macroscopic signs of nerve damage, probably due to a traction injury of the constricted nerve.^13,14^ The macroscopic description of the intraseptal tunnel was provided by Styf et al^14^ and Williams et al.^18^ The former^14^ described its localization in the corner between the AIS and the anterior fascia, characterized by a fibrous floor. The latter^18^ described the passage of the SPN within its fascial tunnel in the substance of the AIS (“intraseptal tunnel” variant). Unfortunately, in both studies, the topography of the tunnel and the description of the nerve branches that coursed in the tunnel was not given. Similarly, available anatomic studies mainly focused on the branching pattern, without providing any topographic description of the transition site and intraseptal tunnel.^3,6^

In our series, the tunnel had a fibrous base, whereas the walls and the roof were thin. The thick fibers which are typical of the AIS were less densely represented at the intracompartmental aspect of the tunnel’s walls and absent at the roof, whose fiber pattern, thickness, and elasticity resembled more those of the crural fascia. Because of the macroscopic blending features of AIS and crural fascia at the transition site, only a histologic examination could distinguish the real origin of the tunnel tissue from the AIS rather than the deep fascia, which was not the purpose of the present study.

In our study, the mean tunnel length was 2.63 cm. There were no cases of associated muscle herniation or fascial defect. The high occurrence of the intraseptal tunnel in our series could confirm the hypothesis of Styf et al^13,14^ that short (<3-cm) tunnels are not symptomatic, and therefore probably more frequent than previously reported.

We also assessed the distribution of the nerve to the compartments and the branching pattern. In 2 cases, the main SPN coming from the anterior compartment passed through an intraseptal tunnel, in the other 8 intraseptal variants the main SPN or its branches came from the lateral compartment. This finding is in line with the studies on the SPN entrapment that have excluded the association of the syndrome with the anatomical variant of SPN coursing in the anterior compartment.^10^

In clinical practice, the knowledge of the intraseptal variant and its relationship with the septum, the crural fascia and the apex of the lateral malleolus has operative relevance. The lateral malleolus is often used as a landmark to identify the site of superficialization of the SPN. In anatomical studies, this was reported as an absolute measure, expressed as the distance in cm between the lateral malleolus and the superficialization site of the nerve.^2^ In the present study, we also adjusted the distance between the transition site and the lateral malleolus to the fibular length (Table 2). The nerve superficialized between the distal two-fourths of the fibula (mean ratio 3.79±1.10). Moreover, we recorded the distance of its deep (proximal) and superficial (distal) opening from the AIS ending and apex of the lateral malleolus. Compared to the extraseptal cases, the superficial opening of the intraseptal cases was 1 cm distal and their deep opening about 2 cm proximal to the superficialization site of the extraseptal cases (Table 2). These measures could help localize the transition site of the SPN and any tunnel and intraseptal SPN variant during open surgeries requiring the identification and sparing of the nerve, such as fasciotomies, fibula flaps, tumor and trauma surgery, but also any proximal extension of the anterior, anterolateral, and lateral approach to the ankle.^3,18^ The use of topographic landmarks would be useful to reduce the occurrence of iatrogenic lesion to the SPN, which accounts for 7.7% to 16.7% in the anterior and anterolateral approach and 21% in the lateral.^5,9^

The main limitation of our study was represented by the small sample size. Fifteen legs from 9 cadavers were available for our study. In 6 of them, both legs were dissected, and an asymmetric anatomy was found. Owing to the high anatomic variability of the SPN,^1,3,4^ a wide number of specimens would have been necessary to adequately study the prevalence of the intraseptal variant.^16^ Nevertheless, our study is the first exploratory study focusing on the anatomic and topographic evaluation of the transition site of the SPN. A small sample size was deemed sufficient for this purpose, thus allowing a wise stewardship of the available human body resource. The absence of a histologic study did not allow us to distinguish the precise origin of the tunnel from the AIS or the crural fascia. Such analysis should be considered in further anatomical studies of the intraseptal tunnel.

## Conclusion

In the present anatomic study, we described the SPN course and branching at the intracompartmental and suprafascial levels with particular attention to the transition site. Compared with available literature, we found a higher rate than expected of intraseptal tunnels and SPN variants at the transition site. This finding could support the hypothesis that the intraseptal SPN variant could not only be associated with the SPN entrapment syndrome but also be found in asymptomatic patients. Apart from the SPN entrapment syndrome, the knowledge of the anatomy of the SPN course and intraseptal variant is relevant to avoid iatrogenic lesions during several surgeries and approaches requiring the identification and sparing of the nerve. Future studies are needed to evaluate the real prevalence of the intraseptal tunnel and how to limit the risk of iatrogenic complications associated.

## Supplemental Material

sj-pdf-1-fai-10.1177_10711007211002508 – Supplemental material for The Intraseptal Course of the Superficial Peroneal Nerve: An Anatomic Study Click here for additional data file. Supplemental material, sj-pdf-1-fai-10.1177_10711007211002508 for The Intraseptal Course of the Superficial Peroneal Nerve: An Anatomic Study by Silvia Valisena, Axel Gamulin and Didier Hannouche in Foot & Ankle International
